# Special Societies and the Amalgamation Scheme

**Published:** 1906-05-19

**Authors:** 


### The Hospital

A Journal of

The Medical Sciences and Hospital Administration.

Vol. XL.—No. 1,025. SATURDAY, MAY 19, 1906.

### Special Societies and the Amalgamation Scheme.

The attempt to establish a scheme of amalgamation for the union of the various metropolitan medical societies has promptly produced evidence of the many difficulties which lie in the way of such an ambition. With all possible goodwill there are many qualifying and opposing influences. Traditions extending over many years and rich in many proud and tender associations appeal to a sense of loyalty in the existing generation and oppose a sacrifice of identity on the part of the societies which possess them. Financial interests, although perhaps more susceptible of management, are hardly less imperative in their claim. And, in addition, it is scarcely a matter for surprise that the officials and members of individual societies should fear that the interests which have enjoyed their peculiar care may suffer when placed in charge of a governing authority having, possibly, a wider outlook, but having, also, many other demands to conciliate. This last-mentioned consideration is naturally particularly present to the authorities of those societies devoted to the cultivation of the more special and more recently developed branches of medical science. The feeling is that the sympathy necessary for the vitality and development of such subjects can hardly be expected apart from those whose work and interests are directly concerned, and that amalgamation, by weakening this influence, may have a prejudicial effect. Appeal is made on this point, and not without justification, to the course of history. It is urged that the special subjects, as they may be termed, received at the outset but scant courtesy and the very reverse of encouragement from the staffs of the general hospitals, and that it was only when the pioneers promoted separate institutions that progress was made and due recognition accorded. In view of all this it is not to be wondered at that special interests regard with some anxiety a proposal to remove them, at least in part, from the control of those directly concerned to a government having many other claims on its attention. With this attitude of mind it is impossible to avoid a certain measure of sympathy, and we, personally, should certainly have no enthusiasm for a scheme of amalgamation which would hinder in the slightest degree the progress and development of the work now prosecuted by the special societies. Doubtless specialists, like most other people, have the defects of their qualities; but their work has been, and we doubt not will continue to be, fruitful in results both to the science and the art of medicine. We cannot, however, believe that the difficulties above suggested are insuperable, and when the professional and scientific position of many of those who cultivate special practice is considered, there ought not to be any difficulty in securing such representation as will prevent neglect of, or injustice to, any and every legitimate interest. At this moment it is not our concern to deal with any particular scheme of amalgamation or with the whole question in any broad or general sense. But there does exist in some quarters a definite suspicion that a union of societies will inevitably mean the sacrifice of those having a more limited scope to those which profess a wider appeal. If this suspicion is cherished it will almost certainly prove fatal and will thus, as we think, destroy an opportunity of much scientific value both to general and special interests. At the present moment the isolation of the various societies means the frequent discussion of questions and problems from a single point of view. If in a special society, the contributors necessarily consider the issue from their own special standpoint; whilst in societies concerned with less specialised observations there is often an absence of precise knowledge upon some limited point which, however, may be vital to the general question. What is often wanted is the introduction of another point of view on the one hand and an expert opinion on the other. If amalgamation were accomplished, the agenda papers of all the sections would come under the notice of members concerned with all branches of practice, and it cannot be doubted that the discussions would gain not only in attendance but also in character and value. No true service is rendered to any branch of medical science by shutting it off from the main body and interest of the profession. It is rather by allying itself with broad and general truths and doctrines that specialism finds both its sure defence and its larger opportunity. That a chance of securing these ends in an increasing degree should be lost through what we cannot but consider an ill-grounded fear, would indeed be pitiable. Traditions, vested interests, financial difficulties, may, together or separately, prove insurmountable obstacles to any scheme of amalgamation. But let it not be said that the prospect of enlarging and co-ordinating the various activities of the profession was sacrificed to a gratuitous and unnecessary suspicion.

